# Self supervised learning based emotion recognition using physiological signals

**DOI:** 10.3389/fnhum.2024.1334721

**Published:** 2024-04-09

**Authors:** Min Zhang, YanLi Cui

**Affiliations:** Computer College, Huanggang Normal University, Huanggang, Hubei, China

**Keywords:** emotional recognition, self-supervised learning, physiological signals, representation learning, deep learning

## Abstract

**Introduction:**

The significant role of emotional recognition in the field of human-machine interaction has garnered the attention of many researchers. Emotion recognition based on physiological signals can objectively reflect the most authentic emotional states of humans. However, existing labeled Electroencephalogram (EEG) datasets are often of small scale.

**Methods:**

In practical scenarios, a large number of unlabeled EEG signals are easier to obtain. Therefore, this paper adopts self-supervised learning methods to study emotion recognition based on EEG. Specifically, experiments employ three pre-defined tasks to define pseudo-labels and extract features from the inherent structure of the data.

**Results and discussion:**

Experimental results indicate that self-supervised learning methods have the capability to learn effective feature representations for downstream tasks without any manual labels.

## 1 Introduction

Emotional recognition in humans is considered a research theme spanning multiple fields including neuroscience, psychology, health sciences, and engineering. Emotion recognition models will aid in establishing high-precision emotional recognition systems and developing various derivative applications in emotional understanding and management. With the increasingly rapid development of artificial intelligence, the issue of emotional recognition has also garnered more and more attention. For instance, in the field of human-machine interaction, accurately recognizing human emotional states is a key technology (Jia et al., 2021a).

As early as 1884, Mr. William James, the pioneer of American functional psychology and pragmatist philosophy, provided some elucidation on the definition of emotion. In his view, emotions are merely sensations caused by abnormalities in a part of the body. The cognition of emotion is triggered by physiological changes in humans. No psychological change is not caused by a bodily change, and emotional changes inevitably follow changes in some sensory organ (Jia et al., 2020). Over the past 100 or more years of research, the scientific community still does not have a systematic definition of emotion. Some believe that emotion is a psychological experience that people generate when facing external things. Accompanying this psychological experience will produce a series of physiological changes, such as fluctuations in EEG signals. However, the most fundamental components of emotion have always been unanimously recognized by scholars, which mainly include: (1) When an emotion occurs, there must be some kind of physical change; (2) Emotion is controlled by consciousness; (3) The expression of emotion is actually a self-evaluation of what has already happened. When faced with the same thing, different people will have different feelings and emotional expressions. These three components also imply the direction of emotion recognition research.

Early emotion recognition technologies mainly relied on non-physiological signals produced by the human body, such as voice, gestures, and facial expressions. As emotion recognition is applied in more fields, subjects gradually began to deliberately hide their true emotions in external features such as facial expressions, speech, and behavior, bringing certain challenges to emotion recognition research based on non-physiological signal data. Therefore, researchers began to focus the solution of emotion recognition on physiological signals that are difficult to disguise. According to neuropsychological and psychological research, electroencephalogram (EEG) can not only reflect various electrical activities and functional states of the human brain, but it can also reflect effective information about the emotional state of humans (Zhou et al., 2023), and the activity of the cerebral cortex has a huge impact on the production of emotions (Jia et al., 2022c). However, other physiological signals, such as electrooculogram(EOG), electrocardiogram (ECG), and electromyogram (EMG), are indirect reactions caused by emotions and usually lack reasonable evaluation standards and have lower emotion recognition accuracy (Chanel et al., 2011; Jia et al., 2022b). In comparison, EEG signals have become the main research force for the emotion recognition problem due to their advantages of being easy to collect, high authenticity, and strong reliability. However, existing labeled EEG emotion datasets are generally small in scale, posing challenges for emotion recognition based on EEG signals.

To address these challenges, this paper adopts a self-supervised learning method to perform EEG emotion recognition using a small amount of labeled data. The experiments define pseudo labels using three pre-tasks to extract features from the structure of the data itself. From the experimental results, the self-supervised learning method has the ability to learn effective feature representations for downstream tasks without any manual labels and also shows its potential in the emotion recognition problem. Therefore, improving the classification performance and generalization ability of self-supervised learning methods in the emotion recognition problem remains a challenge worth exploring.

## 2 Related work

The field of Brain-Computer Interfaces (BCIs) has attracted increasing attention from researchers, with the advancement of machine learning and the growing integration across multiple disciplines. Emotional Brain-Computer Interface research methods have also gradually shifted from traditional machine learning techniques toward deep learning.

In the early stages of emotion recognition research, the focus was mainly on using facial expressions, gestures, and voice audio as non-physiological signals for emotion recognition and analysis. Initial studies relied on manually extracted features from voice data, but these features were often too shallow to accurately identify human emotions. With the advent of deep learning, Abdel-Hamid et al. (2014) applied convolutional neural networks to voice-based emotion recognition. To further improve the accuracy of emotion recognition models, Huang et al. (2014) and Mao et al. (2014), employed stacked autoencoders (SAE) before convolutional neural networks to extract emotional features. Trigeorgis et al. (2016) proposed an end-to-end speech emotion recognition system, which combined Long Short-Term Memory (LSTM) networks with convolutional neural networks (Hochreiter and Schmidhuber, 1997), significantly improving the accuracy of emotion recognition.

During its developmental phase, emotion recognition research began incorporating physiological signals. Researchers mostly used traditional supervised learning methods for recognition tasks. For example, Atkinson and Campos (2016) initially extracted features from multi-channel EEG signals and then used Support Vector Machines (SVM) for downstream task classification. Verma and Tiwary (2017) first preprocessed EEG signals using Kernel Principal Component Analysis (KPCA), and then utilized K-nearest neighbors and Radial Basis Function (RBF) based SVMs for classification. Zheng et al. (2014) first extracted differential entropy features from multi-channel EEG data, and then employed deep learning models for training and proposed Hidden Markov Models as an auxiliary method.

Moreover, individual differences have a considerable impact on network training. Thus, research on cross-subject emotion recognition methods is crucial for practical applications. This requires the model to extract common features from data across different subjects to enhance cross-subject performance. Some studies have explored emotion recognition issues for cross-subjects using techniques such as adaptive learning, transfer learning, active learning, multi-source weighted adaptation, feature assessment, and selection (Chung et al., 2011; Zander and Jatzev, 2011; Mühl et al., 2014; Chen et al., 2019; Lan et al., 2019). Over the past few years, the convenience and availability of EEG monitoring devices have significantly increased, thus generating an ever-growing amount of physiological signal data requiring interpretation. Traditionally, supervised learning models have been used for classifying and predicting physiological signals, achieving high performance through extensive labeled datasets. However, acquiring labeled physiological signals incurs high economic and time costs. Noise in the data and the complexity of the human brain also make annotating physiological signals challenging, potentially leading to large disparities in expert annotations or label noise. Therefore, a learning mode that does not depend on manual labels is essential. Researchers are naturally focusing on unsupervised learning methods that do not require any label information. However, traditional unsupervised learning methods do not perform as quantifiably well as supervised learning methods in downstream tasks.

**Figure 1:**
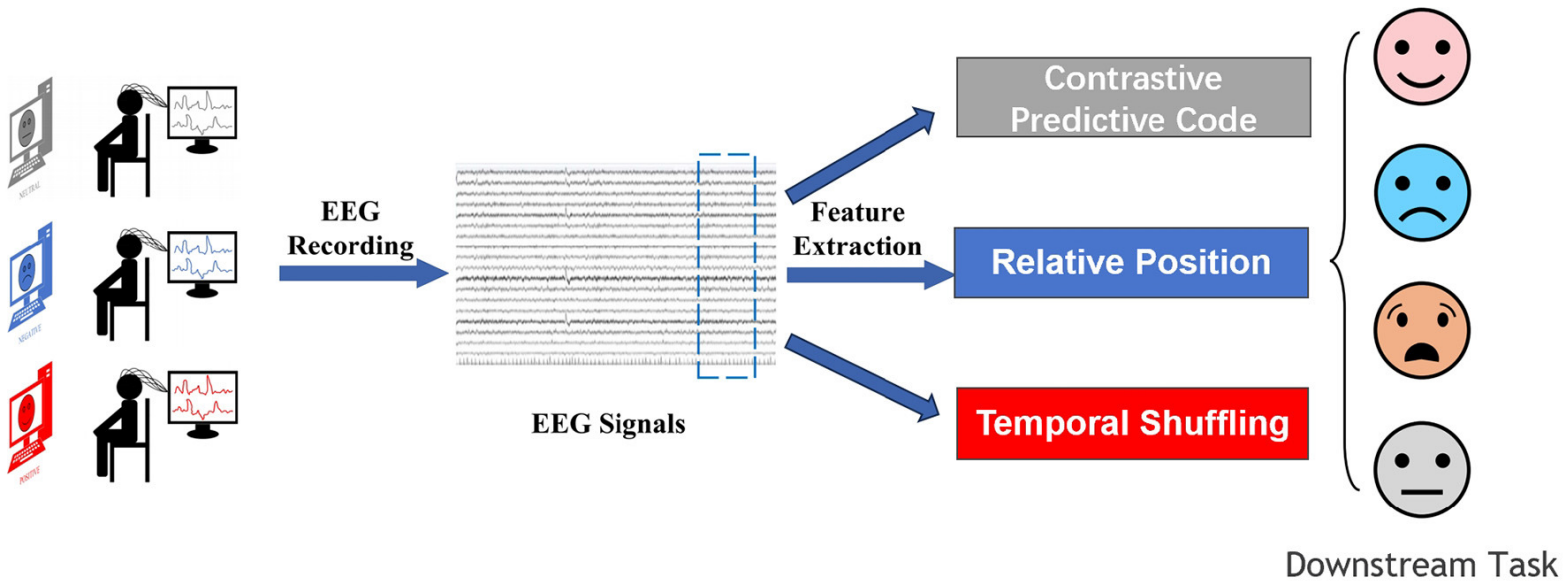
The overall framework in a schematic way.

Self-supervised learning is a form of unsupervised learning that leverages the inherent structure of unlabeled data to provide supervisory signals. Self-supervised learning methods use pretext tasks to reformulate unsupervised learning problems into supervised learning problems, thereby both eliminating the constraint of labels and maintaining the quantifiable advantages of supervised learning. Furthermore, as self-supervised learning relies on the data itself, the features it learns are more universally applicable (Oord et al., 2018). So far, the applications of self-supervised learning have been mostly concentrated in the field of computer vision, where it has become the state-of-the-art in many vision tasks. It has also achieved some success in natural language processing for text classification. However, these fields already have ample labeled data. In contrast, EEG signals, where labeled data are extremely limited, require self-supervised learning methods to solve their classification and prediction issues. Self-supervised learning methods in EEG analysis involve leveraging the inherent structure of electroencephalogram (EEG) data to train models without relying on manual annotations. These approaches typically define auxiliary tasks that exploit temporal and spatial relationships within the EEG signals to generate pseudo-labels. By solving these tasks, the model learns to extract meaningful features that are informative for downstream tasks such as emotion recognition. Unlike supervised methods that require labeled data, self-supervised learning allows for training on large amounts of unlabeled EEG data, making it particularly useful in scenarios where labeled data is scarce or expensive to obtain. Experimental results have shown that self-supervised EEG analysis methods can effectively learn representations that capture the underlying patterns in the data, leading to competitive performance in emotion recognition tasks. Overall, self-supervised learning offers a promising avenue for advancing EEG analysis by enabling models to autonomously discover relevant features and patterns from raw EEG signals as shown in Figure 1.

## 3 Self-supervised learning-based emotional recognition model

In supervised learning models, global optimization problems of neural networks are typically solved using the backpropagation algorithm. The abundance of labeled data and increasingly complex neural network architectures have led to better performance in supervised learning tasks. Therefore, the quality and quantity of labels are key factors determining the efficacy of a model (Jia et al., 2021b; Liu Y. et al., 2023; Liu et al., 2024).

However, in the biomedical field, manually annotated labels are costly and noisy, and annotating large-scale data is time-consuming (Liu S. et al., 2023; Ning et al., 2023). With the development in medical research, it is becoming easier to acquire large volumes of physiological signal data. To break free from the constraints of manual labeling and make full use of physiological signals, researchers have proposed a learning method based on the information within the data itself, known as self-supervised learning (Jaiswal et al., 2020).

As of now, the application of self-supervised learning methods is mostly focused on image, speech, and semantic data. In these fields, sufficient labeled data are available, making supervised learning already highly competitive (Jia et al., 2022a; Liang et al., 2023). In contrast, in areas where labels are hard to obtain, such as physiological signal data, self-supervised learning has greater potential (Krishnan et al., 2022).

Self-supervised learning is an unsupervised learning method but can learn feature representation from unlabeled data, using the structure of the data to provide supervisory information (Zhai et al., 2019). In the field of computer vision, self-supervised learning can extract cropped samples from images using jigsaw puzzles techniques. These samples are then randomly shuffled and fed into a trained neural network to recover the original order. In the temporal domain, self-supervised learning assumes that data changes are continuous and that adjacent time windows correspond to the same label. This is used to extract information from time-series data to predict future frames.

In self-supervised learning, labels are generated through the attributes of the data itself and are called pseudo-labels. These pseudo-labels are produced for the sake of pre-task learning. The method obtains supervisory information from original unlabeled data mainly through pre-tasks to train the network. The trained model is then transferred to downstream tasks. To achieve higher model accuracy, fine-tuning is often required after the parameters have been transferred. This process frees us from the constraints of manual labeling. Therefore, the setting of pre-tasks plays a decisive role in the effectiveness of self-supervised learning and is key to its success (Jaiswal et al., 2020). Table 1 presents the algorithm of self-supervised emotion recognition.

**Table 1:** The algorithm of the self-supervised emotion recognition.

**Step**	**Description**
1	Preprocessing of physiological signals
2	Data segmentation and feature extraction
3	Transforming generative model problems into classification problems
3.1	Train CPC model to maximize mutual information (Equation 1)
3.2	Use log-bilinear model (Equation 2)
3.3	Fit training objective (Equation 3)
4	Generating labeled samples through Relative Position
4.1	Sample *N* samples given hyperparameters τ_*pos*_, τ_*neg*_ (Equation 4)
4.2	Generate labels *y*_*i*_ using time index pairs $\left(t_{i},t_{i}^{\prime }\right)$ (Equation 5)
4.3	Aggregate features using *g*_*RP*_, calculate absolute difference (Equation 6)
4.4	Predict labels *y*_*i*_ using linear discriminant model (Equation 7)
5	Temporal shuffling
5.1	Construct temporally ordered and shuffled triples (Equation 8)
5.2	Feature combination through absolute difference (Equation 9)
5.3	Obtain loss function for temporal shuffling (Equation 10)

### 3.1 Contrastive predictive code

Contrastive predictive code (CPC) is a method for unsupervised learning on high-dimensional data by transforming generative modeling problems into classification problems. The primary aim of the model is to learn feature representations that encode the foundational shared information between different segments of high-dimensional signals while discarding lower-level, less important details. One of the challenges in predicting high-dimensional data is that commonly used loss functions like Mean Squared Error (MSE) and Cross-Entropy are generally ineffective. Therefore, the CPC model is trained by maximizing Mutual Information (MI). Mutual Information is often used to represent the reduction in uncertainty of one random variable due to the knowledge of another, as shown in Equation (1):


(1) $$\begin{array}{l} I\left(x;c\right)=\sum_{x,c}p\left(x,c\right)\log\left(\frac{p\left(x|c\right)}{p\left(x\right)}\right)=H\left(x\right)-H\left(x|c\right) \end{array}$$

Here, *H*(*x*) denotes the entropy of the event *x*, and *c* represents the context vector. *I*(*x*; *c*) signifies the reduction in entropy of *x* due to the introduction of *c*. Thus, maximizing the mutual information between *x* and *c* is equivalent to reducing the uncertainty of predicting *x* to the greatest extent, achieving an improved prediction accuracy.

**Figure 2:**
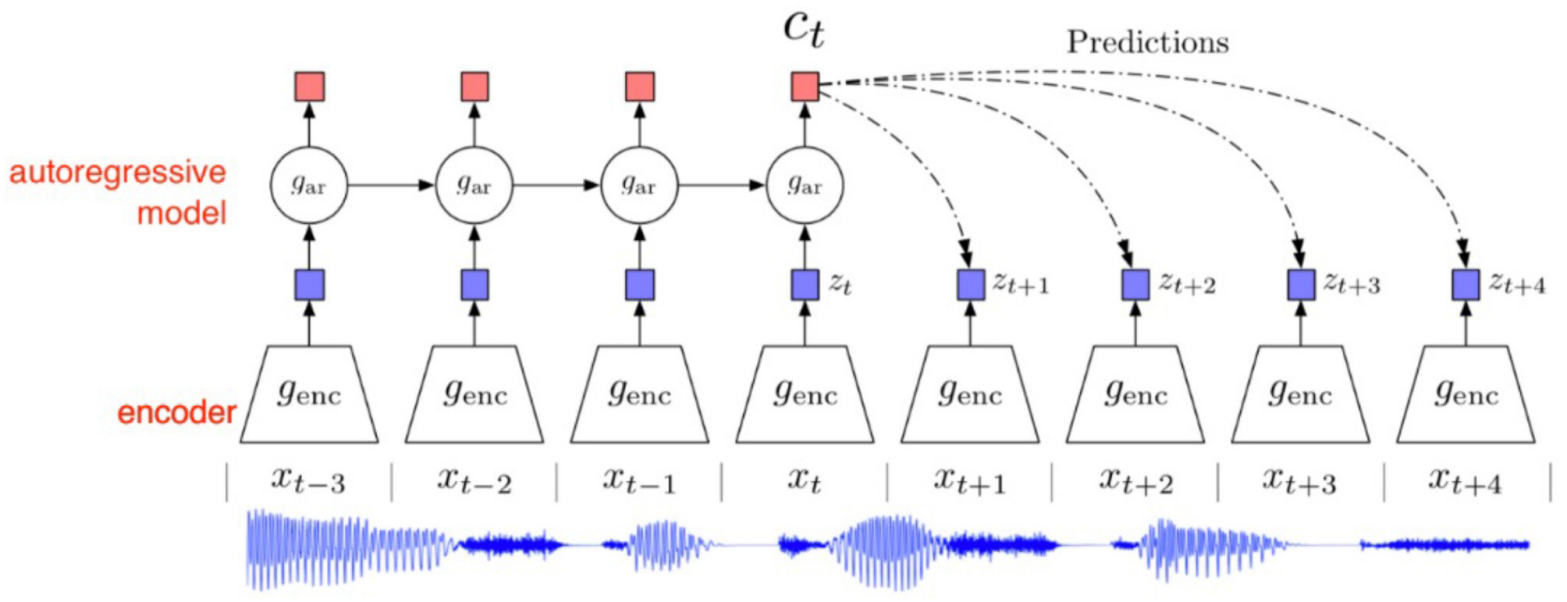
Contrastive predictive model architecture diagram.

Figure 2 depicts the architecture of the Contrastive Predictive Code model. Taking an audio signal as an example, a non-linear encoder *g*_*enc*_ first maps each *x*_*t*_ within a time window to a representation *z*_*t*_ = *g*_*enc*_(*x*_*t*_). The *z*_*t*_ along with the related information from previous moments in the latent space is then input into the autoregressive model *g*_*ar*_, producing the context representation for the current moment *c*_*t*_ = *g*_*ar*_(*z*_≤*t*_). When predicting *z*_*t*+*k*_ k moments later using the current context *c*_*t*_, a function *f*_*k*_(*x*_*t*+*k*_, *c*_*t*_) is proposed to denote the similarity between the predicted ẑ_*t*+*k*_ from *c*_*t*_ and the actual value *x*_*t*+*k*_. This should be proportional to the ratio of the probability of the actual future value *x*_*t*+*k*_ to the probability of a randomly chosen data point: $\frac{p\left(x_{t+k}|c_{t}\right)}{p\left(x_{t+k}\right)}$. We model *f*_*k*_(*x*_*t*+*k*_, *c*_*t*_) using a log-bilinear model as:


(2) $$\begin{array}{l} f_{k}\left(x_{t+k},c_{t}\right)=\exp\left(z_{t+k}^{T}W_{k}c_{t}\right) \end{array}$$

Regarding the training objective, the model adopts the following loss function:


(3) $$\begin{array}{l} L_{N}=-E_{X}\left[\log\left(\frac{f_{k}\left(x_{t+k},c_{t}\right)}{\sum_{x_{j}\in X}f_{k}\left(x_{j},c_{t}\right)}\right)\right] \end{array}$$

where *X* = {*x*_1_, *x*_2_, ..., *x*_*N*_} is a set of samples. The pair (*x*_*t*+*k*_, *c*_*t*_) can be viewed as a positive pair, while any pair (*x*_*j*_, *c*_*t*_) where *j* ≠ *t* + *k* is a negative pair. Thus, maximizing the loss function is equivalent to maximizing the mutual information between positive samples while minimizing the mutual information between negative samples, aligning with the training objectives of the model.

### 3.2 Relative position

For any integers *p, q* ∈ ℕ, let [*q*] denote the set {1, 2, …, *q*}, and [*p, q*] represent the set {*p*, …, *q*}. Let *T* be the time index of a multivariate time series *S* ∈ ℝ^*C*×*M*^, where *M* is the number of time samples and *C* is the dimension of each sample. Let *y* ∈ {−1, 1} be the binary label for the training task.

To generate labeled samples from the multivariate time series *S*, this method samples pairs of time windows $\left(x_{t},x_{t^{\prime }}\right)$, where $x_{t},x_{t^{\prime }}\in {\mathbb{R}}^{C\times T}$, and *T* denotes the duration of each time window. The first window *x*_*t*_ is called the “anchor window.” Assuming that a reasonable feature representation changes slowly over time, adjacent windows should have the same labels. Based on this assumption, given the hyperparameter τ_*pos*_ ∈ ℕ that controls the duration of positive samples and τ_*neg*_ ∈ ℕ for negative samples around each anchor window *x*_*t*_, we sample *N* samples:


(4) $$\begin{array}{l} Z_{N}=\left\{\left(x_{t_{i}},x_{t_{i}^{\prime }}\right),y_{i}|i\in \left[N\right],\left(t_{i},t_{i}^{\prime }\right)\in T,y_{i}\in Y\right\} \end{array}$$

where *Y* ∈ {−1, 1}, and *T* is defined as


$$T\in \left\{\left(t,t^{\prime }\right)\in {\left[M-T+1\right]}^{2}||t-t^{\prime }|\leq \tau_{pos}\text{or}|t-t^{\prime }|>\tau_{neg}\right\}$$

### 3.3 Temporal shuffling

The temporal shuffling (TS) method is a variant of the relative position in the previous section. Two anchor windows *x*_*t*_ and $x_{t^{\prime \prime }}$ are sampled from the positive context. Additionally, a third window $x_{t^{\prime }}$ is sampled either between the two anchor windows or from the negative context. Based on the position of the third window, temporally ordered triplets (*t* < *t*′ < *t*″) and temporally shuffled triplets (*t* < *t*″ < *t*′) or (*t*′ < *t* < *t*″) are constructed. The label at this point is defined based on whether the three-time windows follow the order *t* < *t*′ < *t*″, that is:


(5) $$\begin{array}{l} y_{i}=\{\begin{array}{ll} 1, & \text{if }t<t^{\prime }<t^{\prime \prime }\\ -1, & \text{if }t>t^{\prime }\text{ or }t^{\prime }>t^{\prime \prime } \end{array} \end{array}$$

The contrastive model *g*_*TS*_ is defined as $g_{TS}:{\mathbb{R}}^{D}\times {\mathbb{R}}^{D}\times {\mathbb{R}}^{D}\to {\mathbb{R}}^{2D}$, which also implements feature combination through element-level absolute differences:


$$\begin{array}{l} g_{TS}\left(h_{\theta }\left(x\right),h_{\theta }\left(x^{\prime }\right),h_{\theta }\left(x^{\prime \prime }\right)\right)=\left(\left|h_{\theta }\left(x\right)-h_{\theta }\left(x^{\prime }\right)|,|h_{\theta }\left(x^{\prime }\right)-h_{\theta }\left(x^{\prime \prime }\right)\right|\right) \end{array}$$

(6) $$\begin{array}{l} \in {\mathbb{R}}^{2D} \end{array}$$

Replacing *g*_*RP*_ with *g*_*TS*_ in Equations (6–9) and introducing $x_{t^{\prime \prime }}$, we obtain the loss function for the pre-task Temporal Shuffling:


$$\begin{array}{l} L\left(\Theta ,\omega ,\omega_{0}\right)=\underset{\left(x_{t},x_{t^{\prime }},x_{t^{\prime \prime }},y\right)\in Z_{N}}{\sum } \end{array}$$

(7) $$\begin{array}{l} \log\left(1+\exp\left(-y\left[\omega^{T}g_{TS}\left(h_{\theta }\left(x\right),h_{\theta }\left(x^{\prime }\right),h_{\theta }\left(x^{\prime \prime }\right)\right)+\omega_{0}\right]\right)\right) \end{array}$$

## 4 Experiment

### 4.1 Dataset introduction

#### 4.1.1 SEED dataset

The SEED dataset collected EEG data from fifteen participants, including seven males and eight females, with an average age of ~23 years. During the experiment, participants' emotions were elicited by watching video clips. The emotion labels are defined as positive, neutral, and negative emotions, with five different clips assigned to each emotion. All participants underwent three EEG data recordings, with a 2-week interval between consecutive experiments (Duan et al., 2013). Each time, participants were asked to watch 15 video clips, each about four min long, to induce emotions. The same 15 clips were used across all three recording sessions. Thus, the dataset contains 15 physiological signals for each participant from each recording, resulting in 45 physiological signal datasets per participant after three sessions. Each physiological signal was recorded using a 62-channel ESI NeuroScan device with a sampling rate of 1,000 Hz, down-sampled to 200 Hz. There are three labels in the dataset corresponding to the three emotions (Zheng and Lu, 2015).

#### 4.1.2 SEED-IV dataset

Prior to the experiment, the researchers carefully selected 72 video clips corresponding to four emotion labels: happiness, sadness, fear, and neutral. Similar to the SEED dataset, 15 participants took part in the SEED-IV dataset collection. Each participant attended the experiment at three different times, watching 24 video clips in each session. Each video clip lasted about 120 s, with a 5-s preparation time before each clip and a 45-s self-assessment period after each clip.

#### 4.1.3 DEAP dataset

The DEAP dataset is an emotion dataset collected by the University of Twente. Unlike the SEED dataset, this dataset includes multimodal EEG data, comprising EEG, EMG, and EOG signals (Koelstra et al., 2012). The DEAP dataset includes data from 32 participants, half of whom are male and half female. The experimental protocol is similar to SEED. During the experiment, each participant watched 60-s music videos to elicit emotions. Each EEG recording began with a 3-s preparation period, followed by the 60-s video clip during which emotional EEG data was collected. After the video playback, participants were asked to self-assess their feelings of Valence, Arousal, Dominance, Liking, and Familiarity based on their initial reactions. During the experiment, a short break was organized after watching 20 videos to check the signal quality and inspect the equipment, ensuring the quality of the collected EEG data. The EEG signals in the DEAP dataset were collected using a 32-channel electrode cap, with a sampling rate set to 512 Hz.

Before the experiment, data preprocessing was conducted to extract the time-frequency features of the raw data using short-time Fourier transform. The data were divided into five frequency bands, and differential entropy (DE) features were computed separately for each frequency band. All physiological signals were sampled at 200 Hz. Each record in the dataset was segmented into 1-s data segments. Evaluation was performed using 10-fold cross-validation. The main hardware of the experimental platform is GPU NVIDIA 3090, which has 24 GB of memory. Python for deep learning and Numpy 1.22.3 for numerical computation. The specialized EEG analysis library MNE Python was used for data processing. These configurations contribute to the computational process of EEG data processing, feature extraction, and subsequent emotion classification tasks in the context of self supervised learning paradigms.

Accuracy (ACC): accuracy is the most intuitive performance measure in classification problems. It is the ratio of the number of correct predictions to the total number of predictions. Mathematically, it is expressed as:


(8) $$\begin{array}{l} \text{ACC}=\frac{\text{Number of correct predictions}}{\text{Total number of predictions}}=\frac{TP+TN}{TP+TN+FP+FN} \end{array}$$

where *TP* is true positives, *TN* is true negatives, *FP* is false positives, and *FN* is false negatives.

F1 Macro Score: the F1 Macro Score is a common metric in multi-class classification. It calculates the F1 score for each class individually and then computes the arithmetic mean of these scores. The F1 score is the harmonic mean of precision and recall. For a single class, the F1 score is defined as:


(9) $$\begin{array}{l} F1=2\times \frac{\text{Precision}\times \text{Recall}}{\text{Precision}+\text{Recall}}=\frac{2TP}{2TP+FP+FN} \end{array}$$

The F1 Macro Score is then the average of the F1 scores for all classes:


(10) $$\begin{array}{l} \text{F1 Macro Score}=\frac{1}{N}\overset{N}{\underset{i=1}{\sum }}F1_{i} \end{array}$$

where *N* is the number of classes, and *F*1_*i*_ is the F1 score for the *i*^*th*^ class.

### 4.2 Feature extraction

For the collected EEG signals, it is typically necessary to extract relevant features that are effective for downstream tasks. These extracted features then serve as the basis for subsequent learning and classification tasks. Therefore, the feature extraction stage plays a significant role in EEG signal emotion recognition studies. Extracting appropriate emotion features provides key support for the downstream classification tasks.

Due to the plethora of EEG signal features, effective features are often mixed with some irrelevant or redundant ones. These extraneous features tend to increase computational overhead and can negatively impact the model's generalization capability. Thus, even after feature extraction, feature selection remains necessary. In the initial stages of EEG signal research, researchers typically classified based on the power spectral density feature. As research has deepened, an increasing number of feature extraction methods have been introduced. Common ones include differential entropy, asymmetry difference, asymmetry ratio, and anterior-posterior electrode ratio. In this study, we use the most widely applied and effective feature—differential entropy, also known as the DE feature. Division of frequency bands: δ (1–4 Hz), θ (4–8 Hz), α (8–13 Hz), β (13–30 Hz), γ (30–100 Hz).

Entropy, a concept borrowed from physics, is commonly used in statistics to measure the uncertainty of a random variable *X*. The Shannon entropy *H*(*X*) is calculated as shown in Equation (11), where *p*(*x*) represents the probability of event *x*, and *I*(*x*) = log_2_(*p*(*x*)):


(11) $$\begin{array}{l} H\left(X\right)=\overset{M}{\underset{x=1}{\sum }}p\left(x\right)I\left(x\right)=-\overset{M}{\underset{x=1}{\sum }}p\left(x\right)\log_{2}\left(p\left(x\right)\right) \end{array}$$

While Shannon entropy is applied to discrete variables, for continuous EEG signals, differential entropy is introduced to calculate their complexity as shown in Equation (12).


(12) $$\begin{array}{l} h\left(X\right)=-\int_{X}^{t}f\left(x\right)\log\left(f\left(x\right)\right)dx \end{array}$$

where *f*(*x*) is the probability density function of the random variable *X*.

Given that EEG signals divided into specific frequency bands essentially follow a Gaussian distribution [21], by substituting the probability density function $f_{X\sim N\left(\mu ,\sigma^{2}\right)}\left(x\right)=\frac{1}{\sqrt{2\pi \sigma }}e^{-\frac{{\left(x-\mu \right)}^{2}}{\sigma^{2}}}$ into the above equation, we obtain the calculation formula for the EEG signal DE feature as shown in Equation (13):


$$\begin{array}{l} h\left(X\right)=-\overset{1}{\underset{X}{\int }}\frac{1}{\sqrt{2\pi \sigma }}e^{-\frac{{\left(x-\mu \right)}^{2}}{\sigma^{2}}}\log\left(\frac{1}{\sqrt{2\pi \sigma }}e^{-\frac{{\left(x-\mu \right)}^{2}}{\sigma^{2}}}\right)dx \end{array}$$

(13) $$\begin{array}{l} =\frac{1}{2}\log\left(2\pi e\sigma^{2}\right) \end{array}$$

### 4.3 Results analysis

To verify whether the self-supervised learning method is genuinely applicable to EEG data to achieve the purpose of eliminating the constraints of manual labels, we applied the results of pre-training of the previous task to three emotion datasets (SEED, SEED-IV, and DEAP), and compared their performance in downstream tasks with the supervised learning method described earlier. To control experimental variables, we used the CNN network from previous experiments as the feature extractor and trained using different methods (CPC, RP, TS) to extract features from unlabeled data. The experimental results are shown in Table 2. The baseline percentages for SEED, SEED-IV, and DEAP were 33.3%, 25.0%, and 25.0%, respectively. We have transformed DEAP into a four classification task based on the common data processing methods used in the past. Specifically, we have chosen Valence and Arousal as the two main dimensions of emotional research. These numerical data values range from 0 to 10. Therefore, 5 is usually used as a threshold to binarize each dimension. Therefore, DEAP was transformed into a four class task with a baseline of 25%.

**Table 2:** SSL classification results in downstream emotion recognition tasks.

		**RP**	**TS**	**CPC**	**Baseline**
SEED	Accuracy/Std (%)	35.49/0.43	35.91/1.46	51.90/4.63	33.33
	F1_macro/Std (%)	34.41/1.27	35.11/1.69	50.12/5.75	33.33
SEED-IV	Accuracy/Std (%)	28.51/1.02	29.64/1.05	33.21/4.34	25.00
	F1_macro/Std (%)	27.89/1.06	28.59/0.08	30.89/4.51	25.00
DEAP	Accuracy/Std (%)	52.76/5.08	50.11/4.65	55.21/6.62	25.00
	F1_macro/Std (%)	48.89/2.82	48.27/5.66	44.83/4.86	25.00

From the results in Table 3, we can clearly see that there are distinct differences in the effectiveness of the features extracted by the three pre-tasks when used for classification. In the SEED dataset, the RP pre-task in the three-way classification experiment is equivalent to having no classification ability. However, the CPC method shows significant potential. The accuracy of the CPC method in the three-way classification problem can reach 51.90%, indicating that the self-supervised learning method has the capability to learn useful representational features for downstream tasks without any manual labels. For different datasets, different pre-tasks demonstrate varied effects in downstream emotion recognition tasks. Overall, the CPC method is relatively stable across the three datasets.

**Table 3:** SSL classification time in downstream emotion recognition tasks.

		**RP**	**TS**	**CPC**
SEED	s/epoch	1.59	1.60	1.58
SEED-IV	s/epoch	1.32	1.31	1.30
DEAP	s/epoch	1.49	1.35	1.31

In this paper, we propose to apply the self-supervised learning method, which does not require manual labels for learning, to the emotion recognition problem. By defining labels for the original data through three pre-tasks: Relative Position, Temporal Shuffling, and Contrastive Predictive Code, we learn feature representations through pre-training from the data itself. Through experimental results, we can see that different pre-tasks have distinct classification effects on downstream tasks. The results of the Contrastive Predictive Code method indicate that the self-supervised learning method can learn useful representational features for downstream tasks without any manual labels. Comparisons of results between RP, TS, and CPC were subjected to Wilcoxon pairwise tests. All combinations were corrected for multiple testing, indicating the validity of the conclusions. The purpose of the test is to demonstrate the effectiveness of the CPC method in improving results. RP vs. CPC (*p*-values = 0.001). TS vs. CPC (*p*-values = 0.001). We provide more details on the computational time required to apply the entire self supervised learning framework on each dataset as shown in Table 3.

## 5 Limitations and future directions

Integrating model interpretability into the framework is indeed a crucial aspect, especially in domains like neuroscience where understanding the underlying neural mechanisms is essential. The ability to discern between meaningful neurophysiological features and irrelevant artifacts is paramount for ensuring the reliability and validity of the decoding process, particularly in tasks such as emotion classification.

The approach of incorporating *ad-hoc* interpretable elements into neural networks (Borra et al., 2019, 2022, 2023b; Zhao et al., 2019), as explored in studies like those by Borra et al., represents a promising direction. By designing networks with built-in mechanisms for identifying relevant spatial and frequency neural signatures, researchers can enhance the interpretability of the model's decisions. These interpretable elements not only facilitate understanding the model's inner workings but also aid in identifying which features contribute most significantly to the decoding task.

Moreover, the utilization of deep learning frameworks equipped with model explainability techniques (Schirrmeister et al., 2017; Lawhern et al., 2018; Farahat et al., 2019; Vahid et al., 2020; Borra and Magosso, 2021; Borra et al., 2021, 2023a) such as saliency maps, layerwise relevance propagation, and SHapley Additive exPlanations (SHAP) further enhances the interpretability of neural network models. These methods provide insights into how the model arrives at its predictions, offering valuable clues about which input features are influential in driving the decision-making process.

In the future development of our framework, we acknowledge the importance of integrating interpretability techniques to enhance the transparency and trustworthiness of the decoding process. By incorporating methods like those mentioned above, we aim to provide neuroscientists with not only accurate decoding results but also meaningful insights into the neural substrates underlying the observed phenomena. This approach will not only improve the interpretability of our framework but also foster greater collaboration and understanding between machine learning and neuroscience communities.

## 6 Conclusion

Over the past few decades, emotion recognition, due to its crucial role in the field of human-computer interaction, has always been favored by researchers. Meanwhile, with the development of artificial intelligence, neuroscience has received unprecedented attention. EEG signals, because of their objectivity and accuracy, have gradually been introduced into the field of emotion recognition. This paper primarily bases its research on EEG signals and explores different emotion recognition methods on multiple emotion datasets. Considering the cost and reliability of manually labeled EEG signals, this paper proposes the application of a self-supervised learning method for emotion recognition that doesn't require manual labels. Labels are defined for the original data through three pre-tasks: Relative Position, Temporal Shuffling, and Contrastive Predictive Code, and feature representations are learned through pre-training from the data itself. Through the experimental results, we can see that different pre-tasks have distinct classification effects on downstream tasks. The results of the Contrastive Predictive Code method indicate that the self-supervised learning method can learn useful representational features for downstream tasks without any manual labels.

## Data availability statement

The original contributions presented in the study are included in the article/supplementary material, further inquiries can be directed to the corresponding author.

## Author contributions

MZ: Conceptualization, Data curation, Formal analysis, Funding acquisition, Investigation, Methodology, Project administration, Resources, Software, Supervision, Validation, Visualization, Writing – original draft, Writing – review & editing. YC: Formal analysis, Investigation, Methodology, Project administration, Software, Visualization, Writing – original draft, Writing – review & editing.
